# Electric Field Tuning Non-volatile Magnetism in Half-Metallic Alloys Co_2_FeAl/Pb(Mg_1/3_Nb_2/3_)O_3_-PbTiO_3_ Heterostructure

**DOI:** 10.1186/s11671-018-2489-2

**Published:** 2018-03-06

**Authors:** Gesang Dunzhu, Fenglong Wang, Cai Zhou, Changjun Jiang

**Affiliations:** 1 Key Laboratory for Magnetism and Magnetic Materials of MOE, Lanzhou University, Lanzhou, 730000 People’s Republic of China; 2 Key Laboratory of Special Function Materials and Structure Design, Ministry of Education, Lanzhou University, Lanzhou, 730000 People’s Republic of China

**Keywords:** Heusler alloys, Co_2_FeAl/Pb(Mg_1/3_Nb_2/3_)O_3_-PbTiO_3_ heterostructures, Piezostrain, Non-volatile, Electric field-mediated

## Abstract

We reported the non-volatile electric field-mediated magnetic properties in the half-metallic Heusler alloy Co_2_FeAl/Pb(Mg_1/3_Nb_2/3_)O_3_-PbTiO_3_ heterostructure at room temperature. The remanent magnetization with different applied electric field along [100] and [01-1] directions was achieved, which showed the non-volatile remanent magnetization driven by an electric field. The two giant reversible and stable remanent magnetization states were obtained by applying pulsed electric field. This can be attributed to the piezostrain effect originating from the piezoelectric substrate, which can be used for magnetoelectric-based memory devices.

## Background

With the rapid development of information technology, the increasing demand for high speed, low power dissipation, and non-volatility in applied devices has been received great attention in recent years. Aiming to meet the need, the electric field control magnetism via magnetoelectric (ME) coupling in the ferromagnetic/ferroelectric (FM/FE) multiferroic heterostructures has been proved to be able to provide a combination of the above advantages. In these FM/FE heterostructures [1–9], ME coupling mechanisms have been widely recognized as three aspects, including piezostrain effect, charge effect, and exchange bias [10–15]. Among this, the piezostrain is obtained by piezostrain effect when the electric field was applied on the ferroelectric material, which can induce a large magnetic response of magnetic layer. Based on the piezostrain-mediated ME coupling, the particular ferroelectric crystal material Pb(Mg_1/3_Nb_2/3_)O_3_-30%PbTiO_3_(PMN-PT) with a large piezostrain effect is often used in FM/FE heterostructure, because the *d*_33_ of the material is much larger than the *d*_31_; strain or charge induced by electric field applied to the PMN-PT layer can manipulate the magnetic anisotropy of the adjacent magnetic layer, which results in a ME effect [16–18]. In the FM/FE heterostructure, the half-metallic Heusler alloy Co_2_FeAl (CFA) as the magnetic layer should be used as an eligible material choice [19–22]. The CFA thin film has excellent material properties, such as a low magnetic damping constant, high spin polarization, and a high Curie temperature (1000 K), which are regarded as ideal spin-polarized electron sources for spintronics devices [23, 24]. Wu et. al. reported the piezoelectric strain response in the (011)-oriented single ferroelectric material. The relatively large changes in remannent strain was obtained only applied and released by an electric field [25]. However, the piezostrain-mediated magnetic properties of a magnetic layer by applying an electric field on the PMN-PT substrate are essential for the application in the electronics devices. Therefore, in this paper, we investigated non-volatile electric field-mediated magnetic properties in Co_2_FeAl/Pb(Mg_1/3_Nb_2/3_)O_3_-PbTiO_3_ heterostructure at room temperature. The non-volatile electric-field-driven remanent magnetization along [100] and [01-1] directions was achieved, and the two giant reversible and stable remanent magnetization states are obtained by applying pulsed electric field [26]. This can be attributed to the piezostrain effect originating from the piezoelectric substrate, which can be a potential candidate for electronics devices application.

## Methods

The heterostructure was composed of CFA alloy as FM layer and PMN-PT (011) as FE layer. CFA thin film was deposited by direct current (DC) magnetron sputtering at 600 °C under an Ar pressure of 0.1 Pa and flow rate at 10 SCCM (SCCM denotes cubic centimeter per minute at STP), with a base pressure of 2 × 10^−5^ Pa. The thickness of CFA thin film was 40 nm. The Pt layers were sputtered by 2 mm-thickness Pt target as electrodes. The thickness of the top and bottom Pt layer were 10 and 50 nm, respectively. Cu wires were connected to the electrodes by the adhesive tape. The static magnetic properties of the CFA/PMN-PT heterostructure were investigated by vibrating sample magnetometer (VSM, MicroSense EV9). The DC power supply (Keithley 2410) was used to provide biased voltage. The magnetic domain images were recorded by magnetic force microscopy (MFM) using Asylum Research© MFP-3D at room temperature with soft magnetic tips magnetized perpendicularly to the sample plane. All the measurements were conducted at room temperature.

## Results and Discussions

**Fig. 1:**
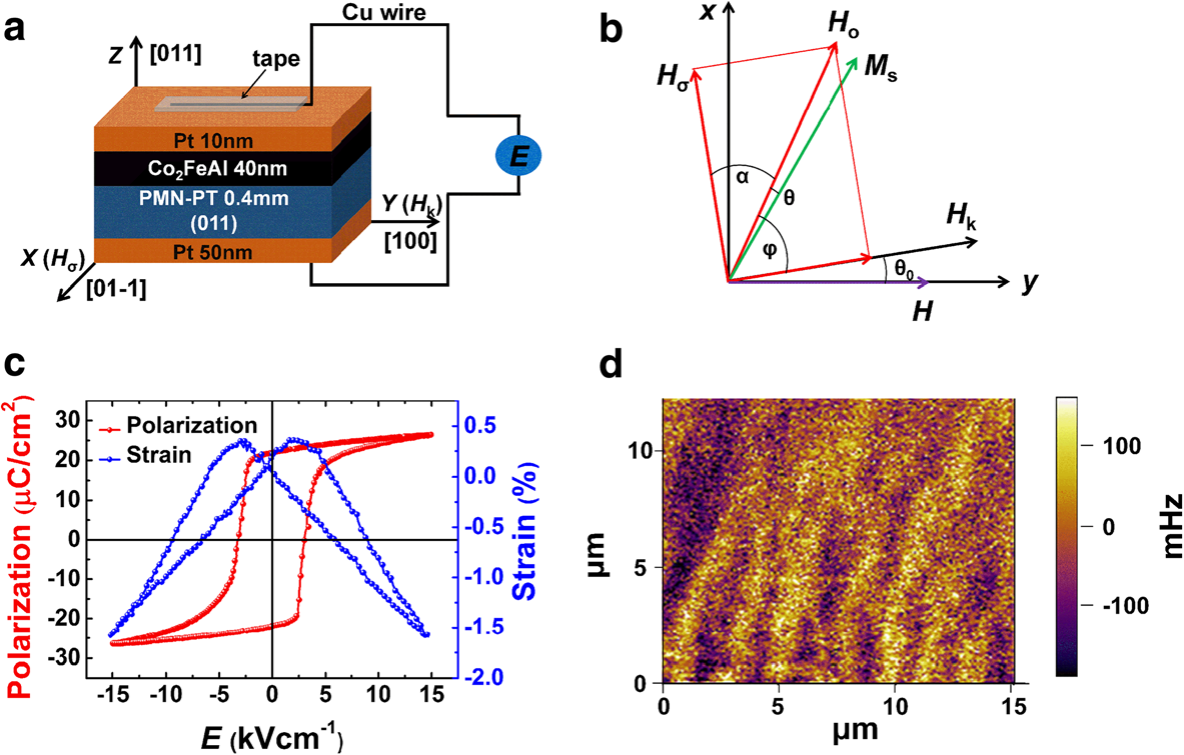
Schematic of the CFA/PMN-PT multiferroic heterostructure (**a**) and schematic of the coordinate system (**b**). *α*, *φ*, and *θ* are the angles of the effective electric-field-induced piezostrain field (*H*_σ_), magnetic anisotropy field (*H*_k_), and magnetization (*M*_s_) with respect to the total effective field (*H*_0_), respectively. *θ*_0_ is the angle of the *H*_k_ with respect to the magnetic field (*H*). **c** The hysteresis loop (*P*-*E* loop, 1 Hz) and strain curve (*S*-*E*) of PMN-PT substrate along [100] direction. **d** A typical MFM image of CFA film

The basic building blocks of the CFA/PMN-PT heterostructure and the coordinate system of in-plane static magnetic measurement were shown in Fig. 1a, b, respectively. The effective electric-field-induced piezostrain field (*H*_σ_) and magnetic anisotropy field (*H*_k_) are perpendicular to each other. We define the magnetic field *H* applied along [100] direction as 0°, whereas, the [01-1] direction as 90° [26]. From the PMN-PT hysteresis loop (*P*-*E* loop, 1 Hz) and strain curve (*S*-*E*), which measured by ferroelectric and strain gauges in Fig. 1c, we can see that the saturation polarization of PMN-PT is about 25 μCcm^−2^, and the coercive field is about 100 V (2.5 KVcm^−1^). MFM image is measured when the applied magnetic field 1000 Oe was removed as shown in Fig. 1d. The dark and light areas indicate the formation of an out-of-plane magnetization component. Consequently, an array of oscillating “up and down” magnetic domain forms, known as stripe domain (SD), which suggests the existence of sizeable perpendicular magnetic anisotropy [27].

**Fig. 2:**
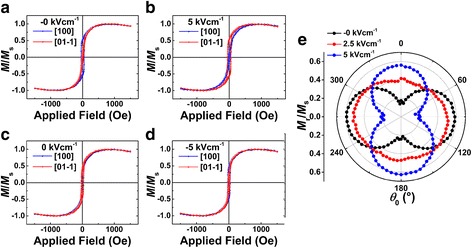
**a–d** The magnetic hysteresis loops at − 0, 0, 5, and − 5 kVcm^−1^, respectively. **e** Measured *M*_r_/*M*_s_ versus *θ*_0_ curves under various electric fields

The magnetic hysteresis loops of the CFA/PMN-PT heterostructure were measured along the direction of [100] and [01-1] under applied electric fields of ± 0 and ± 5 kVcm^−1^ [11]. The electric field was applied from the top to the bottom as positive, otherwise negative. The − 0 and + 0 kVcm^−1^ are remnant polarization states after the applied electric fields of − 10 and + 10 kVcm^−1^ turned off, respectively. The magnetic hysteresis loops as shown in Fig. 2a indicated a clear in-plane magnetic anisotropy. The blue line represents easy magnetization direction of in-plane hysteresis loop along the direction [100], and the remanent magnetization is significantly smaller than the saturation magnetization. The *M*-*H* loops were constituted by a two magnetization process: the *M*-*H* curve exhibits a linear relationship between the applied magnetic field from the positive saturation field to the negative coercivity field and the abrupt reverse of *M* when the *H* reaches coercivity field; the *M*-*H* curve returns to linear relationship as the applied magnetic field continues to decrease, which can be considered that the film has a stripe domain structure. The red line denotes hard magnetization direction, which is measured along the direction [01-1]. Figure 2b shows the hysteresis loops of the CFA/PMN-PT heterostructure under the electric field *E* = 5 kVcm^−1^. Compared with the result as shown in Fig. 1a, easy axis direction rotates 90°, that is to say, it is rotating from the direction [100] to [01-1] [28–30]. As shown in Fig. 2c, the blue line coincides with the red line, and the in-plane magnetic anisotropy disappears under the polarization state + 0 kVcm^−1^. The magnetic easy axis returns to [100] direction when the applied electric field continues to decrease to − 5 kVcm^−1^ as shown in Fig. 2d. In order to investigate the change of the magnetic anisotropy field with different electric fields, the remanent magnetization at different angles was measured as shown in Fig. 2e. In this measurement, the sample was rotated from 0° to 360° in the plane with the step of 5°. The in-plane magnetic anisotropy is measured in the CFA/PMN-PT heterostructure. At − 0 kVcm^−1^, the easy magnetization direction of in-plane remanent magnetization curve is along the direction [100]. The value of relative remanent magnetization (*M*_r_/*M*_s_) is significantly smaller than 1, which indicates a part of the magnetic moment not coherent arrangement. With increasing electric field to + 2.5 kVcm^−1^, the magnetic anisotropy decreases. When continuing to increase the electric field to + 5 kVcm^−1^, the in-plane magnetic anisotropy reappears. Compared with the remanent magnetization curve at − 0 and + 5 kVcm^−1^, the easy axis rotates 90°, which is consistent with the result of hysteresis loops as shown in Fig. 2a, b. This can be attributed to the piezostrain effect induced by electric field, and the piezoelectric effect of PMN-PT will produce new magnetic anisotropy (stress anisotropy *H*_σ_) in the CFA/PMN-PT heterostructure. The magnetic anisotropy of the CFA/PMN-PT heterostructures is affected by the combination of *H*_σ_ and *H*_k_ [31].

**Fig. 3:**
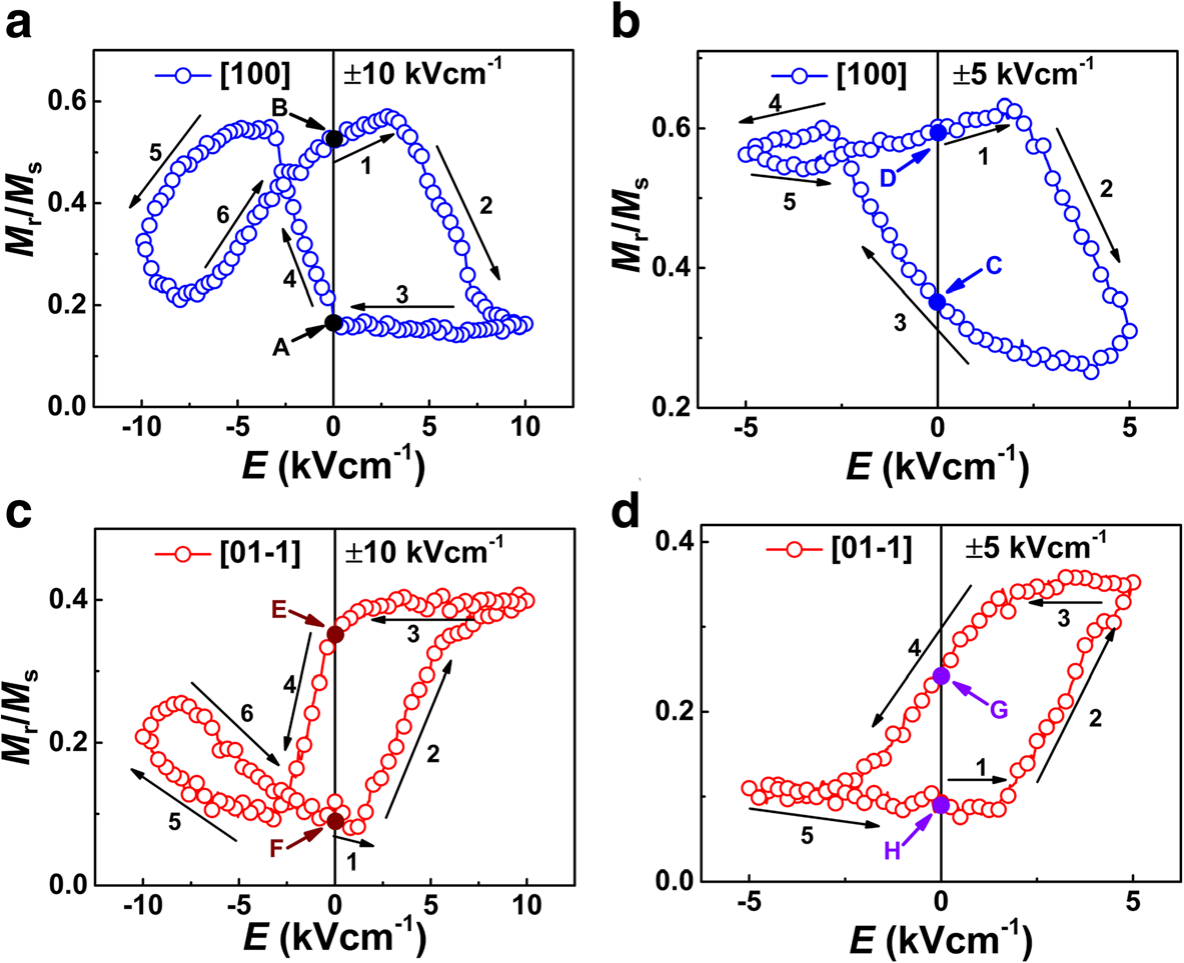
**a**, **c** The dependence of *M*_r_/*M*_s_ of the electric field was measured by sweeping the electric field form + 10 to − 10 kVcm^−1^ in the [100] and [01-1] directions, respectively. **b**, **d** The dependence of *M*_r_/*M*_s_ on the electric field was measured by sweeping the electric field from form + 5 to − 5 kVcm^−1^ in the [100] and [01-1] directions, respectively. The numbers and arrows express the steps and direction of the measurement. And the capital letters in this figure express the values of *M*_r_/*M*_s_ at the remnant polarization states

In order to verify the piezostrain effect induced by the electric field, the remanent magnetization with the applied electric field in the [01-1] and [100] directions was measured. We measured the change of remanent magnetization by sweeping the electric field after removing the saturation magnetic field 1200 Oe in the [100] and [01-1] directions, respectively. The asymmetric butterfly-like remanent magnetization versus applied electric field is obtained. We can determine that the remanence of the CFA/PMN-PT heterostructure is responsive to an electric field shaped as a butterfly; the *M*-*E* curves were measured by sweeping the electric field from + 10 to − 10 kVcm^−1^ in Fig. 3a, c. This response is symmetrical with the variation curve of stress with electric field, which indicates that the stress effect plays a dominant role in the magnetic control of the sample. It is worth noting that the residual magnetization in the remnant polarization state (± 0 kVcm^−1^) is different from + 10 kVcm^−1^ demonstrated by the capital letters A and E in Fig. 3 and − 10 kVcm^−1^ demonstrated by B and F, which is the residual stress from the PMN-PT substrate. Residual polarization state is the remanence of the 0 kVcm^−1^ state, which is derived from the PMN-PT substrate residual stress, and not the same at + 10 and − 10 kVcm^−1^. It is consistent with the residual strain of strain curve in Fig. 1c.

We have carried out experiments on the relationship between the remanence in the unsaturated polarization state (± 5 kVcm^−1^) with the electric field in the [100] and [01-1] directions, in order to reflect the non-volatile control of the electric field. It can be found that the change of the remanence with the electric field also shows a change in the shape as a loop-like, and the remanence of the sample shows a good non-volatile, which is from the remnant polarization stress under the positive and negative electric fields, as shown in Fig. 3b, d. This has a good prospect for stress-tolerant non-volatile memory devices.

**Fig. 4:**
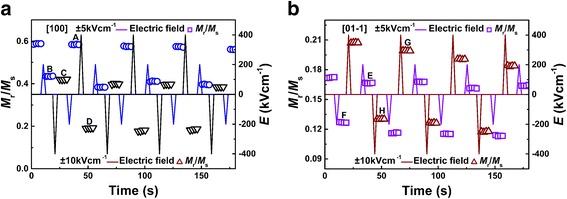
The normalized remnant magnetization ratio *M*_r_/*M*_s_ under the pulsed electric field. **a** The change in *M*_r_/*M*_s_ under the pulsed electric fields ± 5 and ± 10 kVcm^−1^ along [100] direction, respectively. **b** The change in *M*_r_/*M*_s_ under the pulsed electric field ± 5 and ± 10 kVcm^−1^ along [01-1] direction, respectively. The capital letters in this figure express the various remnant polarization states

For magnetic memory application, the non-volatile remanence in the pulsed electric field was achieved, as shown in Fig. 4. Intermittent positive and negative electric fields of ± 5 or ± 10 kVcm^−1^ are applied across the sample in the [100] and [01-1] directions. Firstly, the magnetic field is set up to 1200 Oe and reduced to 0 subsequently. Then the pulsed electric field is first stuck at ± 5 kVcm^−1^ in the [100] direction and reduced to 0 subsequently with results of the two residual polarization states demonstrated by the capital letters A and B in Fig. 4a. The similar case for ± 10 kVcm^−1^ was also observed as other residual polarization states C and D in Fig. 4a, which also reflects the non-volatile states in our sample. When the pulsed electric fields are applied to − 5 or − 10 kVcm^−1^ and reduced to 0 subsequently, we can see that the remanence is relatively large immediately, and when it is applied to 5 or 10 kVcm^−1^ and reduced to 0 subsequently, the remanence is significantly reduced; this phenomenon and the value of *M*_r_/*M*_s_ are consistent with the results of Fig 3a, b. We carried out a similar measurement in the other direction of the sample and got similar results as shown in Fig. 4b. It can be seen that four distinct and stable residual magnetic states are switched by two pulsed electric fields. The four resistive states of E, F, G, and H are generated by the pulsed electric field switching of ± 5 and ± 10 kVcm^−1^ and then instantly removed in the [01-1] direction, respectively. In summary, the remanence of Co_2_FeAl/PMN-PT heterogeneous is stress control and thus realizing the multistate remanence under the pulsed electric field, which can be used for polymorphic storage.

## Conclusions

In summary, the non-volatile electric field-mediated magnetic properties in the CFA/PMN-PT heterostructure are investigated at room temperature. The striped domain structure was obtained by the MFM measurement in the CFA film. The magnetic anisotropy was modulated by the electric field. The result measured by rotating-angle VSM demonstrates piezostrain-mediated non-volatile 90° magnetic easy axis rotation at − 0 and + 5 kVcm^−1^. Additionally, the piezostrain-mediated non-volatile stable remanent magnetization reversal in the two directions is observed under positive and negative pulsed electric fields, which can be used for magnetic storage [32, 33].

## Abbreviations

CFA: Co_2_FeAl
DC: Direct current
FM/FE: Ferromagnetic/ferroelectric
ME: Magnetoelectric
MFM: Magnetic force microscopy
PMN-PT: Pb(Mg_1/3_Nb_2/3_)O_3_-30%PbTiO_3_
VSM: Vibrating sample magnetometer
